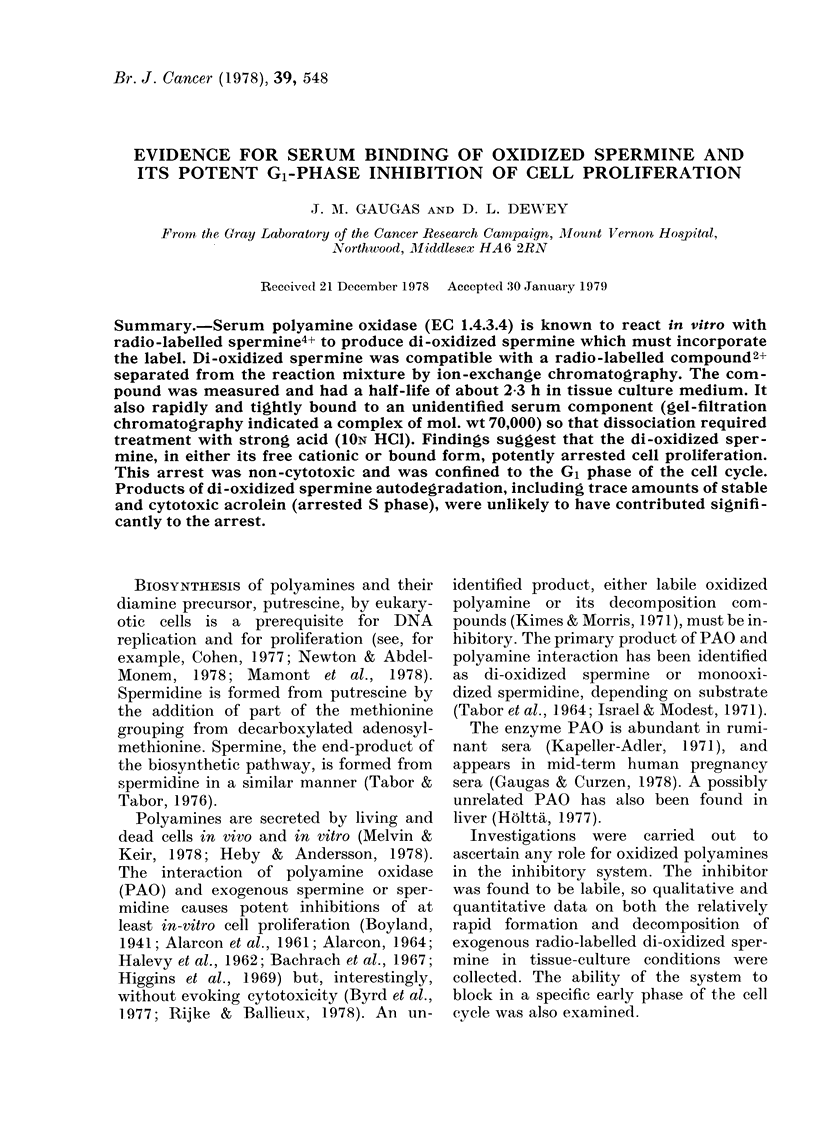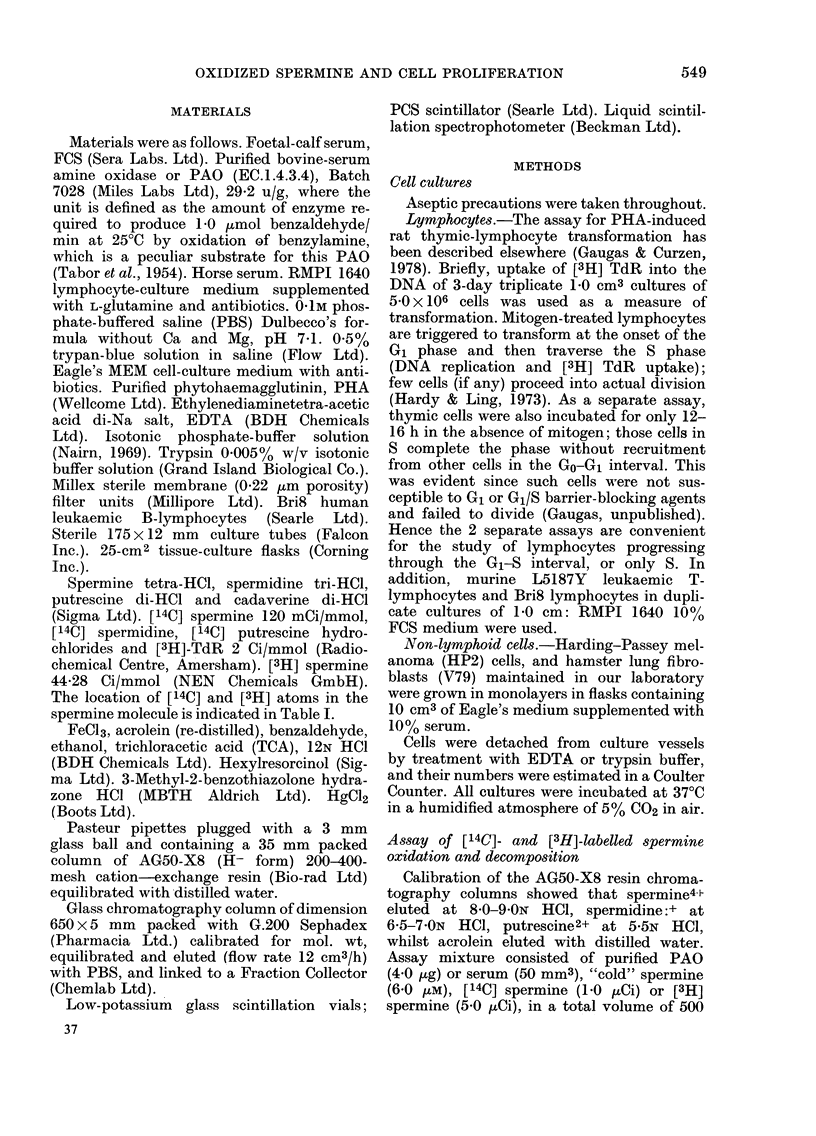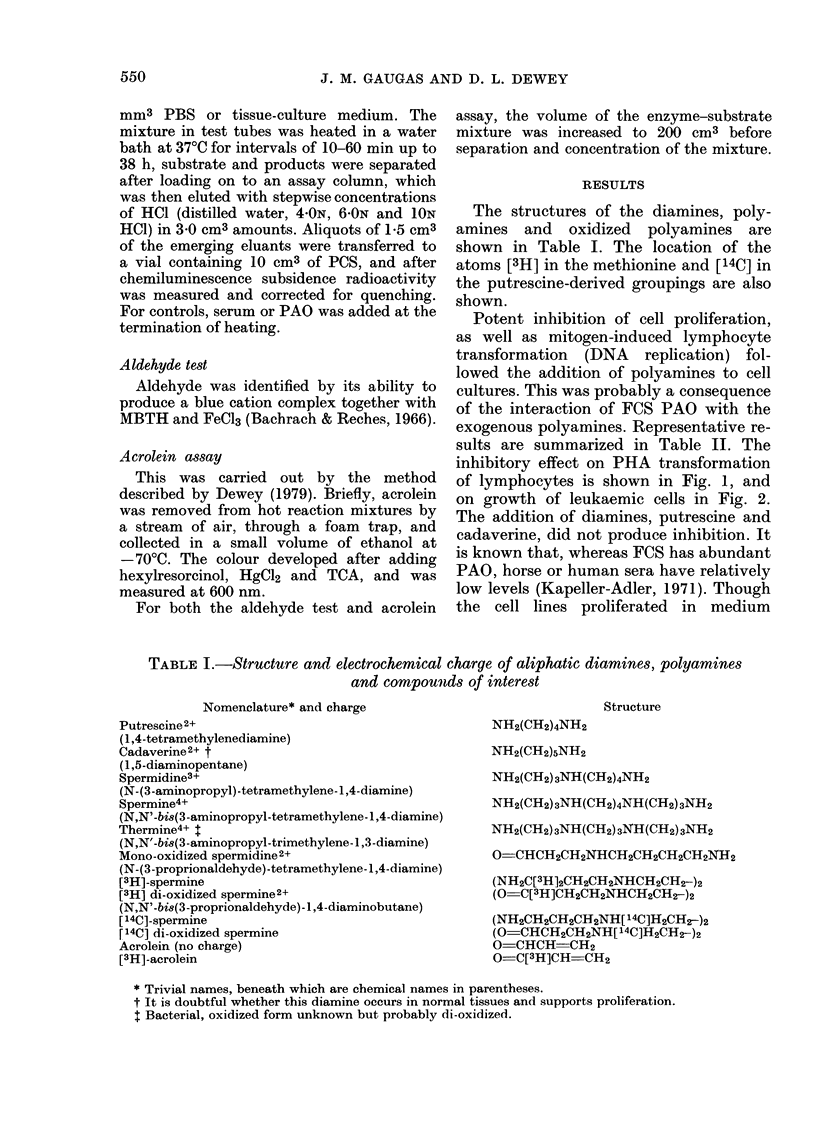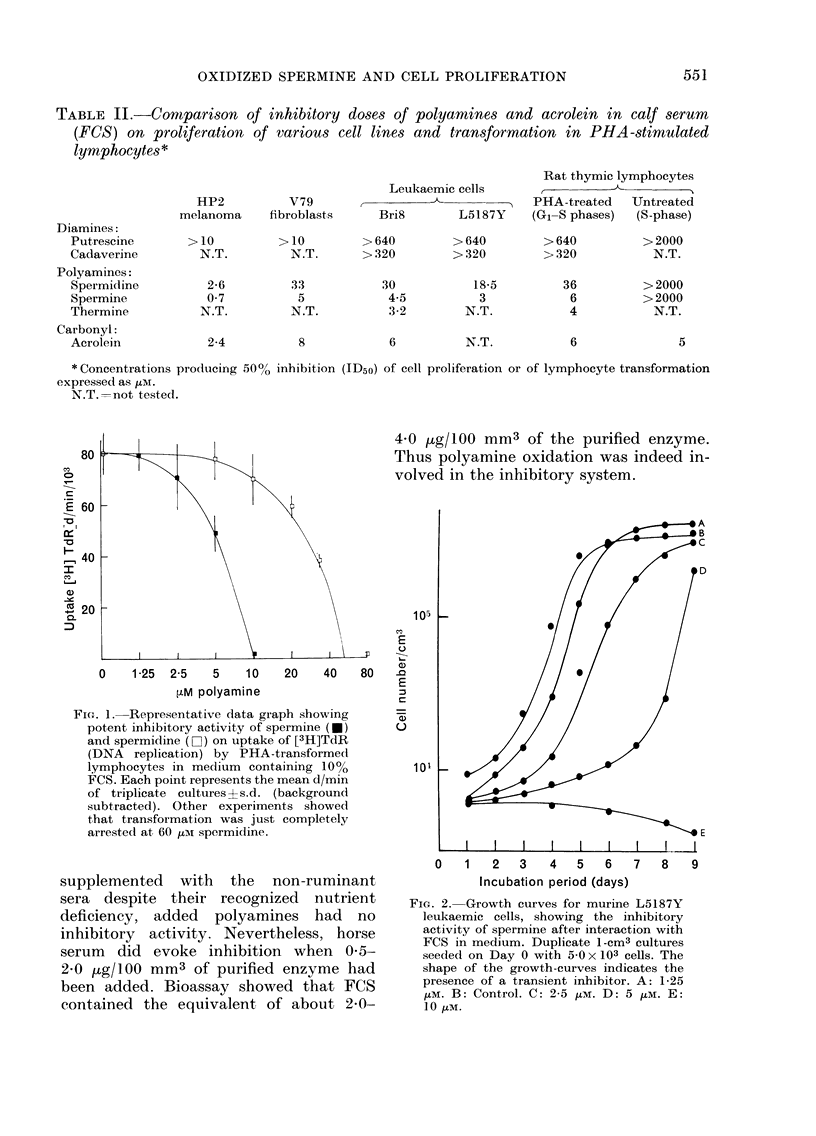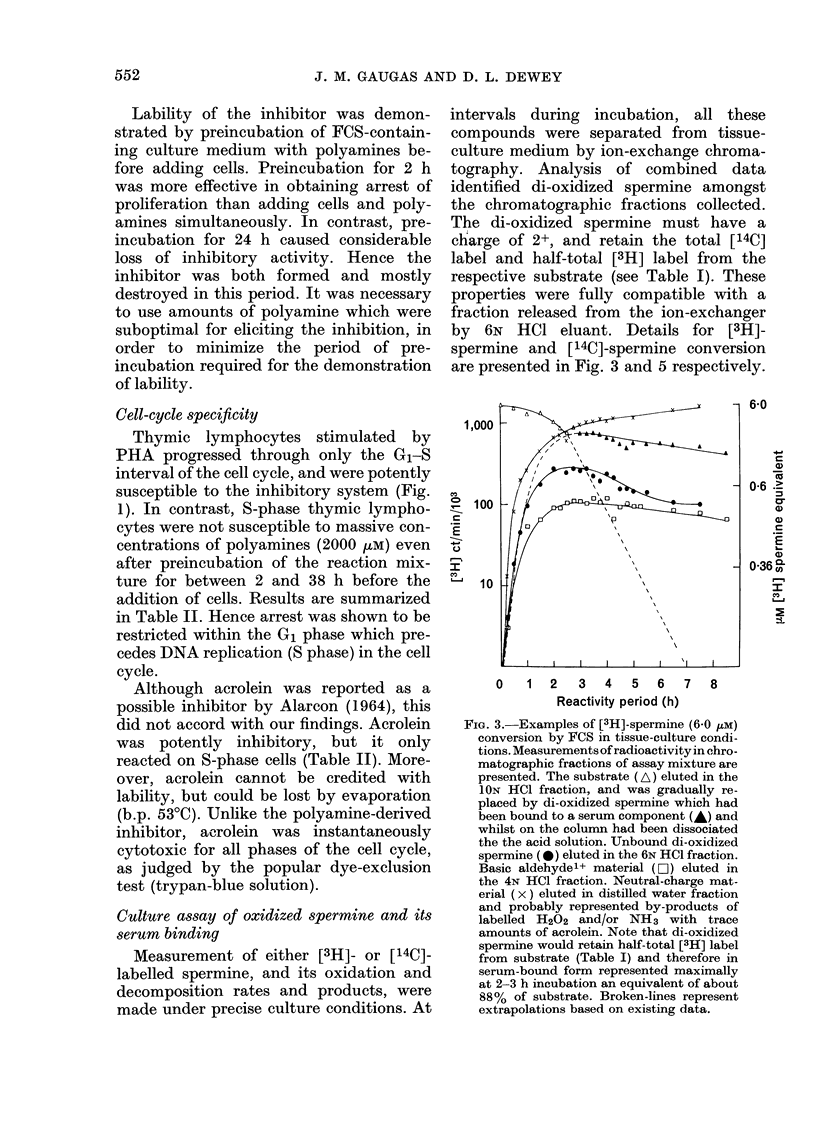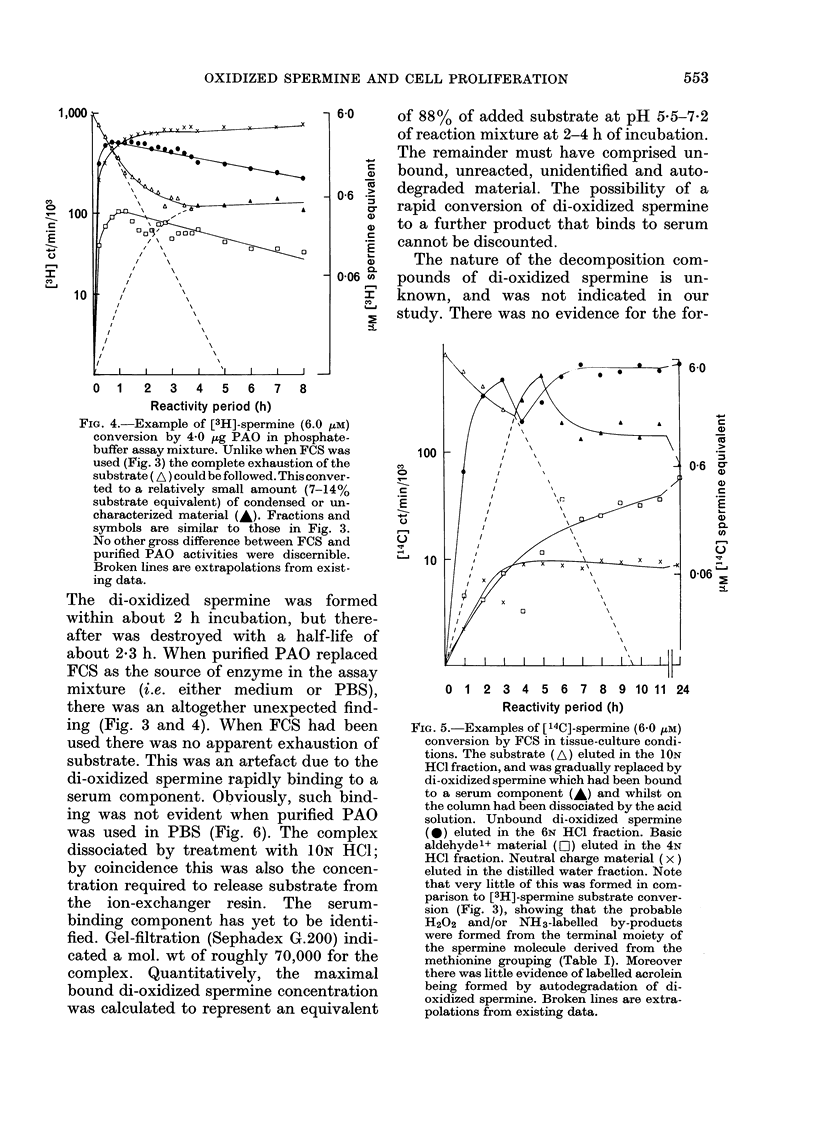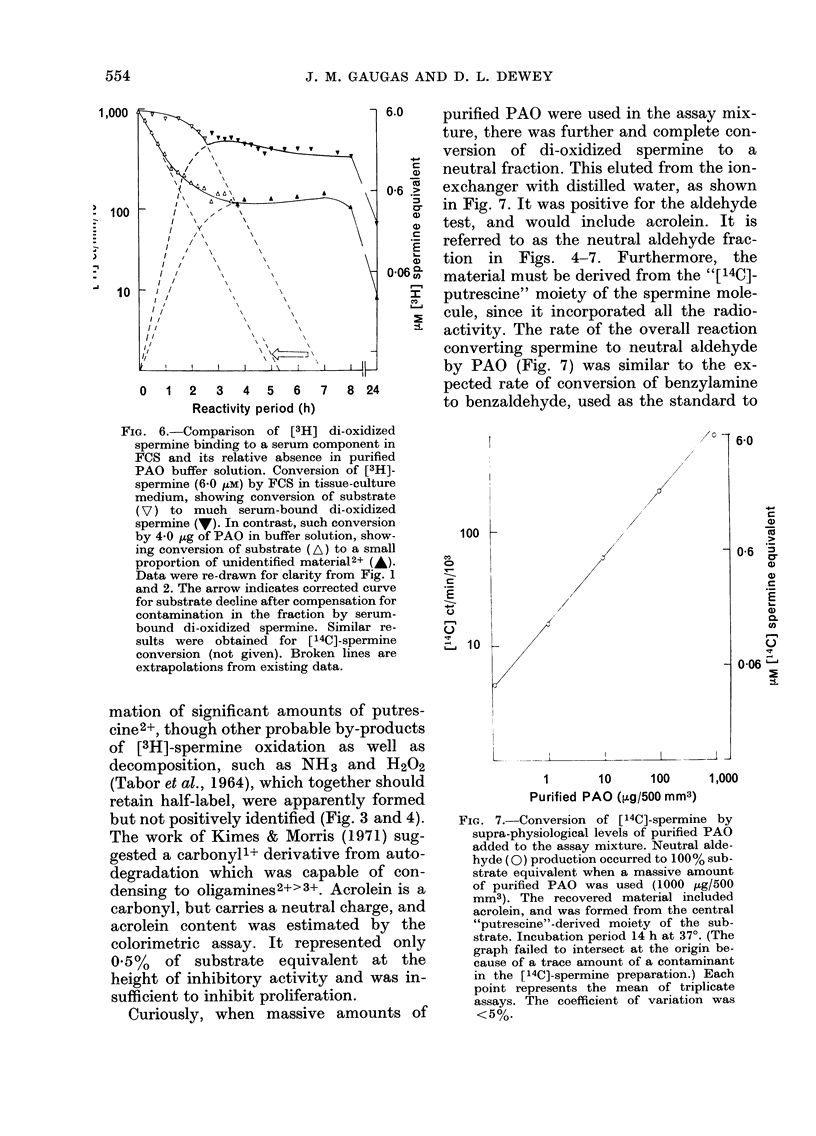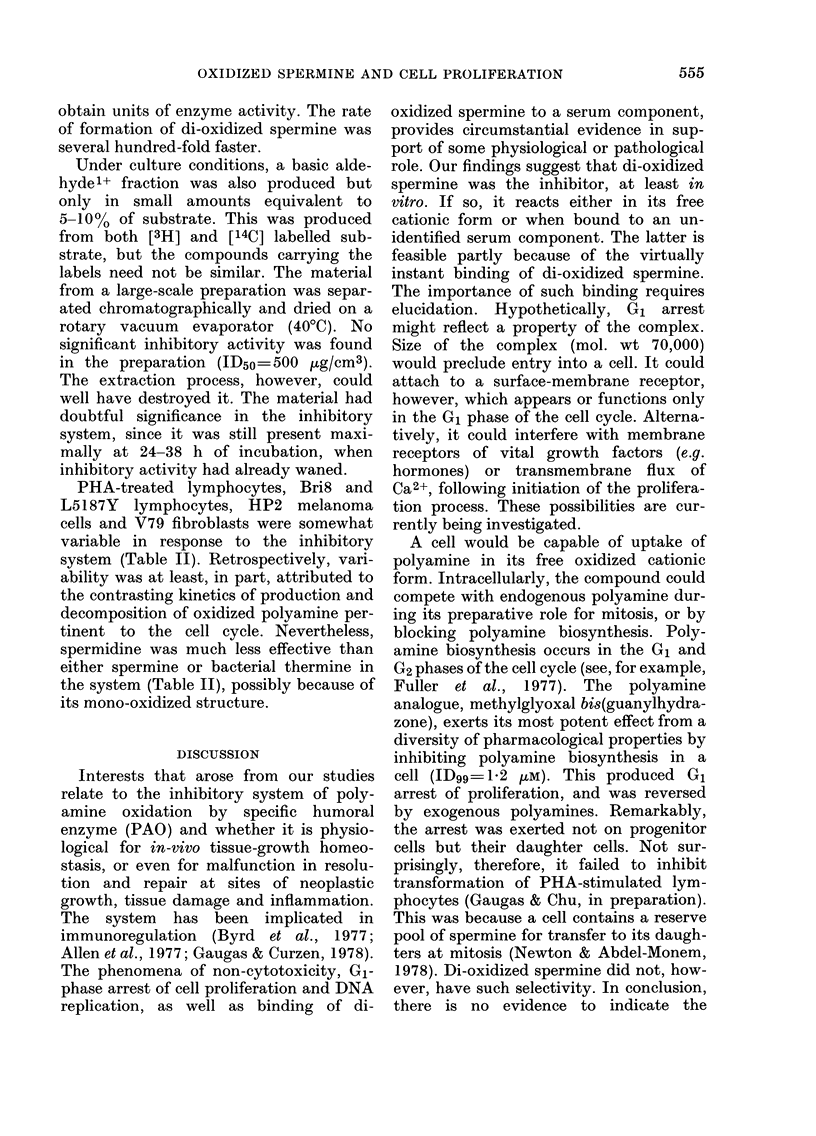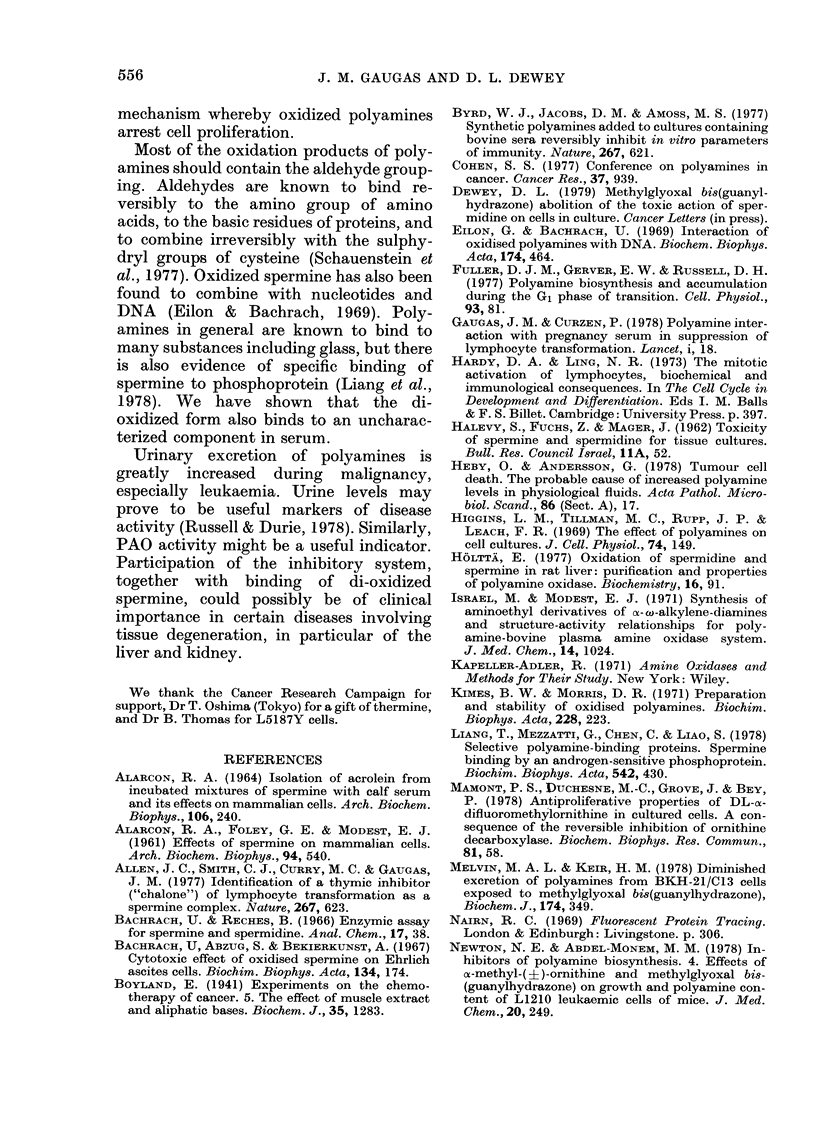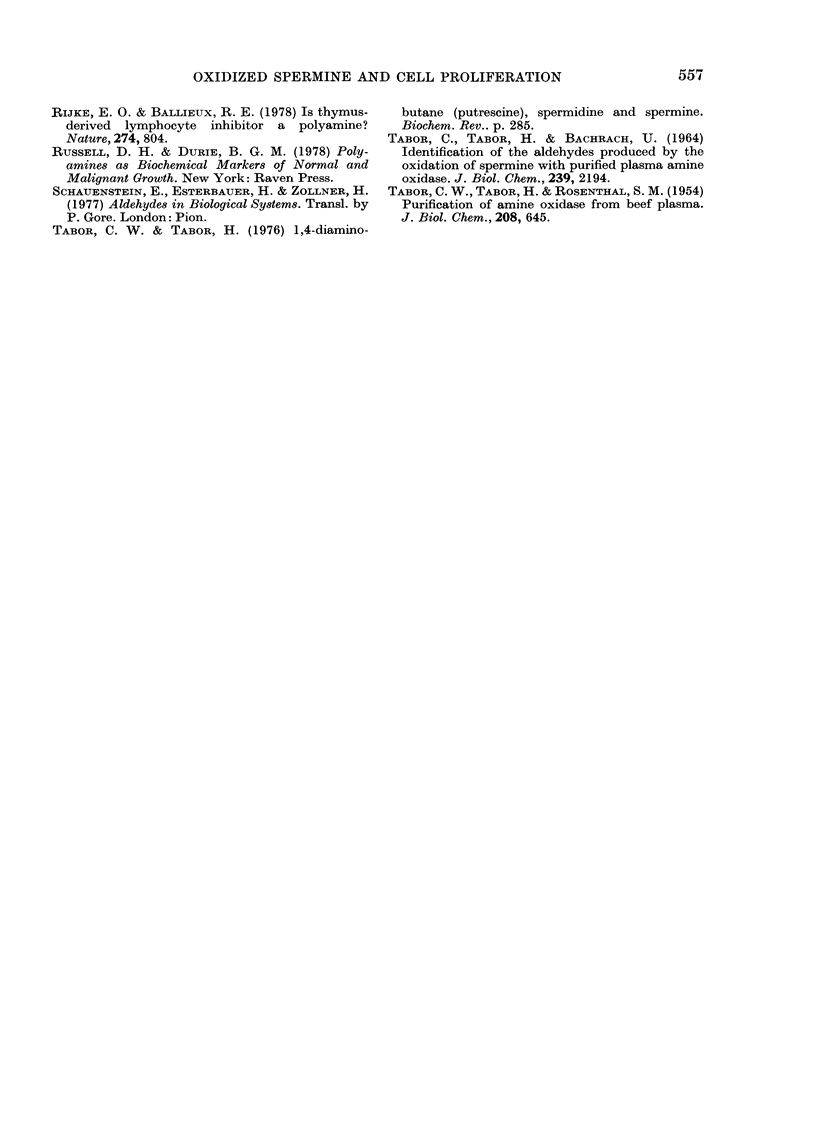# Evidence for serum binding of oxidized spermine and its potent G1-phase inhibition of cell proliferation.

**DOI:** 10.1038/bjc.1979.100

**Published:** 1979-05

**Authors:** J. M. Gaugas, D. L. Dewey

## Abstract

Serum polyamine oxidase (EC 1.4.3.4) is known to react in vitro with radio-labelled spermine4+ to produce di-oxidized spermine which must incorporate the label. Di-oxidized spermine was compatible with a radio-labelled compound2+ separated from the reaction mixture by ion-exchange chromatography. The compound was measured and had a half-life of about 2.3 h in tissue culture medium. It also rapidly and tightly bound to an unidentified serum component (gel-filtration chromatography indicated a complex of mol. wt 70,000) so that dissociation required treatment with strong acid (10N HCl). Findings suggest that the di-oxidized spermine, in either its free cationic or bound form, potently arrested cell proliferation. This arrest was non-cytotoxic and was confined to the G1 phase of the cell cycle. Products of di-oxidized spermine autodegradation, including trace amounts of stable and cytotoxic acrolein (arrested S phase), were unlikely to have contributed significantly to the arrest.


*Br. J. Cancer* (1978), **39**, 548

### EVIDENCE FOR SERUM BINDING OF OXIDIZED SPERMINE AND ITS POTENT $G_1$-PHASE INHIBITION OF CELL PROLIFERATION

J. M. GAUGAS AND D. L. DEWEY

*From the Gray Laboratory of the Cancer Research Campaign, Mount Vernon Hospital, Northwood, Middlesex HA6 2RN*

Received 21 December 1978 Accepted 30 January 1979

**Summary.—Serum polyamine oxidase (EC 1.4.3.4) is known to react *in vitro* with radio-labelled spermine$^{4+}$ to produce di-oxidized spermine which must incorporate the label. Di-oxidized spermine was compatible with a radio-labelled compound$^{2+}$ separated from the reaction mixture by ion-exchange chromatography. The compound was measured and had a half-life of about 2·3 h in tissue culture medium. It also rapidly and tightly bound to an unidentified serum component (gel-filtration chromatography indicated a complex of mol. wt 70,000) so that dissociation required treatment with strong acid (10N HCl). Findings suggest that the di-oxidized spermine, in either its free cationic or bound form, potently arrested cell proliferation. This arrest was non-cytotoxic and was confined to the $G_1$ phase of the cell cycle. Products of di-oxidized spermine autodegradation, including trace amounts of stable and cytotoxic acrolein (arrested S phase), were unlikely to have contributed significantly to the arrest.**

Biosynthesis of polyamines and their diamine precursor, putrescine, by eukaryotic cells is a prerequisite for DNA replication and for proliferation (see, for example, Cohen, 1977; Newton & Abdel-Monem, 1978; Mamont *et al.*, 1978). Spermidine is formed from putrescine by the addition of part of the methionine grouping from decarboxylated adenosylmethionine. Spermine, the end-product of the biosynthetic pathway, is formed from spermidine in a similar manner (Tabor & Tabor, 1976).

Polyamines are secreted by living and dead cells *in vivo* and *in vitro* (Melvin & Keir, 1978; Heby & Andersson, 1978). The interaction of polyamine oxidase (PAO) and exogenous spermine or spermidine causes potent inhibitions of at least *in-vitro* cell proliferation (Boyland, 1941; Alarcon *et al.*, 1961; Alarcon, 1964; Halevy *et al.*, 1962; Bachrach *et al.*, 1967; Higgins *et al.*, 1969) but, interestingly, without evoking cytotoxicity (Byrd *et al.*, 1977; Rijke & Ballieux, 1978). An unidentified product, either labile oxidized polyamine or its decomposition compounds (Kimes & Morris, 1971), must be inhibitory. The primary product of PAO and polyamine interaction has been identified as di-oxidized spermine or monooxidized spermidine, depending on substrate (Tabor *et al.*, 1964; Israel & Modest, 1971).

The enzyme PAO is abundant in ruminant sera (Kapeller-Adler, 1971), and appears in mid-term human pregnancy sera (Gaugas & Curzen, 1978). A possibly unrelated PAO has also been found in liver (Hölttä, 1977).

Investigations were carried out to ascertain any role for oxidized polyamines in the inhibitory system. The inhibitor was found to be labile, so qualitative and quantitative data on both the relatively rapid formation and decomposition of exogenous radio-labelled di-oxidized spermine in tissue-culture conditions were collected. The ability of the system to block in a specific early phase of the cell cycle was also examined.

### MATERIALS

Materials were as follows. Foetal-calf serum, FCS (Sera Labs. Ltd). Purified bovine-serum amine oxidase or PAO (EC.1.4.3.4), Batch 7028 (Miles Labs Ltd), 29·2 u/g, where the unit is defined as the amount of enzyme required to produce 1·0 μmol benzaldehyde/min at 25°C by oxidation of benzylamine, which is a peculiar substrate for this PAO (Tabor *et al.*, 1954). Horse serum. RMPI 1640 lymphocyte-culture medium supplemented with L-glutamine and antibiotics. 0·1M phosphate-buffered saline (PBS) Dulbecco's formula without Ca and Mg, pH 7·1. 0·5% trypan-blue solution in saline (Flow Ltd). Eagle's MEM cell-culture medium with antibiotics. Purified phytohaemagglutinin, PHA (Wellcome Ltd). Ethylenediaminetetra-acetic acid di-Na salt, EDTA (BDH Chemicals Ltd). Isotonic phosphate-buffer solution (Nairn, 1969). Trypsin 0·005% w/v isotonic buffer solution (Grand Island Biological Co.). Millex sterile membrane (0·22 μm porosity) filter units (Millipore Ltd). Bri8 human leukaemic B-lymphocytes (Searle Ltd). Sterile 175×12 mm culture tubes (Falcon Inc.). 25-cm² tissue-culture flasks (Corning Inc.).

Spermine tetra-HCl, spermidine tri-HCl, putrescine di-HCl and cadaverine di-HCl (Sigma Ltd). [$^{14}$C] spermine 120 mCi/mmol, [$^{14}$C] spermidine, [$^{14}$C] putrescine hydrochlorides and [$^{3}$H]-TdR 2 Ci/mmol (Radiochemical Centre, Amersham). [$^{3}$H] spermine 44·28 Ci/mmol (NEN Chemicals GmbH). The location of [$^{14}$C] and [$^{3}$H] atoms in the spermine molecule is indicated in Table I.

$FeCl_3$, acrolein (re-distilled), benzaldehyde, ethanol, trichloracetic acid (TCA), 12N HCl (BDH Chemicals Ltd). Hexylresorcinol (Sigma Ltd). 3-Methyl-2-benzothiazolone hydrazone HCl (MBTH Aldrich Ltd). $HgCl_2$ (Boots Ltd).

Pasteur pipettes plugged with a 3 mm glass ball and containing a 35 mm packed column of AG50-X8 ($H^-$ form) 200–400-mesh cation—exchange resin (Bio-rad Ltd) equilibrated with distilled water.

Glass chromatography column of dimension 650×5 mm packed with G.200 Sephadex (Pharmacia Ltd.) calibrated for mol. wt, equilibrated and eluted (flow rate 12 cm³/h) with PBS, and linked to a Fraction Collector (Chemlab Ltd).

Low-potassium glass scintillation vials; PCS scintillator (Searle Ltd). Liquid scintillation spectrophotometer (Beckman Ltd).

### METHODS

### *Cell cultures*

Aseptic precautions were taken throughout.

*Lymphocytes.*—The assay for PHA-induced rat thymic-lymphocyte transformation has been described elsewhere (Gaugas & Curzen, 1978). Briefly, uptake of [$^{3}$H] TdR into the DNA of 3-day triplicate 1·0 cm³ cultures of $5{\cdot}0\times10^6$ cells was used as a measure of transformation. Mitogen-treated lymphocytes are triggered to transform at the onset of the $G_1$ phase and then traverse the S phase (DNA replication and [$^{3}$H] TdR uptake); few cells (if any) proceed into actual division (Hardy & Ling, 1973). As a separate assay, thymic cells were also incubated for only 12–16 h in the absence of mitogen; those cells in S complete the phase without recruitment from other cells in the $G_0$–$G_1$ interval. This was evident since such cells were not susceptible to $G_1$ or $G_1$/S barrier-blocking agents and failed to divide (Gaugas, unpublished). Hence the 2 separate assays are convenient for the study of lymphocytes progressing through the $G_1$–S interval, or only S. In addition, murine L5187Y leukaemic T-lymphocytes and Bri8 lymphocytes in duplicate cultures of 1·0 cm: RMPI 1640 10% FCS medium were used.

*Non-lymphoid cells.*—Harding–Passey melanoma (HP2) cells, and hamster lung fibroblasts (V79) maintained in our laboratory were grown in monolayers in flasks containing 10 cm³ of Eagle's medium supplemented with 10% serum.

Cells were detached from culture vessels by treatment with EDTA or trypsin buffer, and their numbers were estimated in a Coulter Counter. All cultures were incubated at 37°C in a humidified atmosphere of 5% $CO_2$ in air.

### *Assay of [$^{14}$C]- and [$^{3}$H]-labelled spermine oxidation and decomposition*

Calibration of the AG50-X8 resin chromatography columns showed that spermine$^{4+}$ eluted at 8·0–9·0N HCl, spermidine:$^{+}$ at 6·5–7·0N HCl, putrescine$^{2+}$ at 5·5N HCl, whilst acrolein eluted with distilled water. Assay mixture consisted of purified PAO (4·0 μg) or serum (50 mm³), "cold" spermine (6·0 μM), [$^{14}$C] spermine (1·0 μCi) or [$^{3}$H] spermine (5·0 μCi), in a total volume of 500

$mm^3$ PBS or tissue-culture medium. The mixture in test tubes was heated in a water bath at 37°C for intervals of 10–60 min up to 38 h, substrate and products were separated after loading on to an assay column, which was then eluted with stepwise concentrations of HCl (distilled water, 4·0N, 6·0N and 10N HCl) in 3·0 $cm^3$ amounts. Aliquots of 1·5 $cm^3$ of the emerging eluants were transferred to a vial containing 10 $cm^3$ of PCS, and after chemiluminescence subsidence radioactivity was measured and corrected for quenching. For controls, serum or PAO was added at the termination of heating.

*Aldehyde test*

Aldehyde was identified by its ability to produce a blue cation complex together with MBTH and $FeCl_3$ (Bachrach & Reches, 1966).

*Acrolein assay*

This was carried out by the method described by Dewey (1979). Briefly, acrolein was removed from hot reaction mixtures by a stream of air, through a foam trap, and collected in a small volume of ethanol at −70°C. The colour developed after adding hexylresorcinol, $HgCl_2$ and TCA, and was measured at 600 nm.

For both the aldehyde test and acrolein assay, the volume of the enzyme–substrate mixture was increased to 200 $cm^3$ before separation and concentration of the mixture.

### RESULTS

The structures of the diamines, polyamines and oxidized polyamines are shown in Table I. The location of the atoms [$^3H$] in the methionine and [$^{14}C$] in the putrescine-derived groupings are also shown.

Potent inhibition of cell proliferation, as well as mitogen-induced lymphocyte transformation (DNA replication) followed the addition of polyamines to cell cultures. This was probably a consequence of the interaction of FCS PAO with the exogenous polyamines. Representative results are summarized in Table II. The inhibitory effect on PHA transformation of lymphocytes is shown in Fig. 1, and on growth of leukaemic cells in Fig. 2. The addition of diamines, putrescine and cadaverine, did not produce inhibition. It is known that, whereas FCS has abundant PAO, horse or human sera have relatively low levels (Kapeller-Adler, 1971). Though the cell lines proliferated in medium

TABLE I.—*Structure and electrochemical charge of aliphatic diamines, polyamines and compounds of interest*

| Nomenclature* and charge | Structure |
|---|---|
| Putrescine$^{2+}$ (1,4-tetramethylenediamine) | $NH_2(CH_2)_4NH_2$ |
| Cadaverine$^{2+}$ † (1,5-diaminopentane) | $NH_2(CH_2)_5NH_2$ |
| Spermidine$^{3+}$ (N-(3-aminopropyl)-tetramethylene-1,4-diamine) | $NH_2(CH_2)_3NH(CH_2)_4NH_2$ |
| Spermine$^{4+}$ (N,N'-*bis*(3-aminopropyl-tetramethylene-1,4-diamine) | $NH_2(CH_2)_3NH(CH_2)_4NH(CH_2)_3NH_2$ |
| Thermine$^{4+}$ ‡ (N,N′-*bis*(3-aminopropyl-trimethylene-1,3-diamine) | $NH_2(CH_2)_3NH(CH_2)_3NH(CH_2)_3NH_2$ |
| Mono-oxidized spermidine$^{2+}$ (N-(3-proprionaldehyde)-tetramethylene-1,4-diamine) | $O{=}CHCH_2CH_2NHCH_2CH_2CH_2CH_2NH_2$ |
| [$^3H$]-spermine | $(NH_2C[^3H]_2CH_2CH_2NHCH_2CH_2{-})_2$ |
| [$^3H$] di-oxidized spermine$^{2+}$ (N,N'-*bis*(3-proprionaldehyde)-1,4-diaminobutane) | $(O{=}C[^3H]CH_2CH_2NHCH_2CH_2{-})_2$ |
| [$^{14}C$]-spermine | $(NH_2CH_2CH_2CH_2NH[^{14}C]H_2CH_2{-})_2$ |
| [$^{14}C$] di-oxidized spermine | $(O{=}CHCH_2CH_2NH[^{14}C]H_2CH_2{-})_2$ |
| Acrolein (no charge) | $O{=}CHCH{=}CH_2$ |
| [$^3H$]-acrolein | $O{=}C[^3H]CH{=}CH_2$ |

* Trivial names, beneath which are chemical names in parentheses.
† It is doubtful whether this diamine occurs in normal tissues and supports proliferation.
‡ Bacterial, oxidized form unknown but probably di-oxidized.

TABLE II.—*Comparison of inhibitory doses of polyamines and acrolein in calf serum (FCS) on proliferation of various cell lines and transformation in PHA-stimulated lymphocytes**

| | HP2 melanoma | V79 fibroblasts | Leukaemic cells: Bri8 | Leukaemic cells: L5187Y | Rat thymic lymphocytes: PHA-treated ($G_1$–S phases) | Rat thymic lymphocytes: Untreated (S-phase) |
|---|---|---|---|---|---|---|
| Diamines: | | | | | | |
| Putrescine | >10 | >10 | >640 | >640 | >640 | >2000 |
| Cadaverine | N.T. | N.T. | >320 | >320 | >320 | N.T. |
| Polyamines: | | | | | | |
| Spermidine | 2·6 | 33 | 30 | 18·5 | 36 | >2000 |
| Spermine | 0·7 | 5 | 4·5 | 3 | 6 | >2000 |
| Thermine | N.T. | N.T. | 3·2 | N.T. | 4 | N.T. |
| Carbonyl: | | | | | | |
| Acrolein | 2·4 | 8 | 6 | N.T. | 6 | 5 |

*Concentrations producing 50% inhibition ($ID_{50}$) of cell proliferation or of lymphocyte transformation expressed as μM.
N.T. = not tested.

FIG. 1.—Representative data graph showing potent inhibitory activity of spermine (■) and spermidine (□) on uptake of [$^3$H]TdR (DNA replication) by PHA-transformed lymphocytes in medium containing 10% FCS. Each point represents the mean d/min of triplicate cultures ± s.d. (background subtracted). Other experiments showed that transformation was just completely arrested at 60 μM spermidine.

supplemented with the non-ruminant sera despite their recognized nutrient deficiency, added polyamines had no inhibitory activity. Nevertheless, horse serum did evoke inhibition when 0·5–2·0 μg/100 mm$^3$ of purified enzyme had been added. Bioassay showed that FCS contained the equivalent of about 2·0–4·0 μg/100 mm$^3$ of the purified enzyme. Thus polyamine oxidation was indeed involved in the inhibitory system.

FIG. 2.—Growth curves for murine L5187Y leukaemic cells, showing the inhibitory activity of spermine after interaction with FCS in medium. Duplicate 1-cm$^3$ cultures seeded on Day 0 with $5{\cdot}0 \times 10^3$ cells. The shape of the growth-curves indicates the presence of a transient inhibitor. A: 1·25 μM. B: Control. C: 2·5 μM. D: 5 μM. E: 10 μM.

Lability of the inhibitor was demonstrated by preincubation of FCS-containing culture medium with polyamines before adding cells. Preincubation for 2 h was more effective in obtaining arrest of proliferation than adding cells and polyamines simultaneously. In contrast, preincubation for 24 h caused considerable loss of inhibitory activity. Hence the inhibitor was both formed and mostly destroyed in this period. It was necessary to use amounts of polyamine which were suboptimal for eliciting the inhibition, in order to minimize the period of preincubation required for the demonstration of lability.

*Cell-cycle specificity*

Thymic lymphocytes stimulated by PHA progressed through only the $G_1$–S interval of the cell cycle, and were potently susceptible to the inhibitory system (Fig. 1). In contrast, S-phase thymic lymphocytes were not susceptible to massive concentrations of polyamines (2000 $\mu$M) even after preincubation of the reaction mixture for between 2 and 38 h before the addition of cells. Results are summarized in Table II. Hence arrest was shown to be restricted within the $G_1$ phase which precedes DNA replication (S phase) in the cell cycle.

Although acrolein was reported as a possible inhibitor by Alarcon (1964), this did not accord with our findings. Acrolein was potently inhibitory, but it only reacted on S-phase cells (Table II). Moreover, acrolein cannot be credited with lability, but could be lost by evaporation (b.p. 53°C). Unlike the polyamine-derived inhibitor, acrolein was instantaneously cytotoxic for all phases of the cell cycle, as judged by the popular dye-exclusion test (trypan-blue solution).

*Culture assay of oxidized spermine and its serum binding*

Measurement of either [$^3$H]- or [$^{14}$C]-labelled spermine, and its oxidation and decomposition rates and products, were made under precise culture conditions. At intervals during incubation, all these compounds were separated from tissue-culture medium by ion-exchange chromatography. Analysis of combined data identified di-oxidized spermine amongst the chromatographic fractions collected. The di-oxidized spermine must have a charge of $2^+$, and retain the total [$^{14}$C] label and half-total [$^3$H] label from the respective substrate (see Table I). These properties were fully compatible with a fraction released from the ion-exchanger by 6N HCl eluant. Details for [$^3$H]-spermine and [$^{14}$C]-spermine conversion are presented in Fig. 3 and 5 respectively.

FIG. 3.—Examples of [$^3$H]-spermine (6·0 $\mu$M) conversion by FCS in tissue-culture conditions. Measurements of radioactivity in chromatographic fractions of assay mixture are presented. The substrate (△) eluted in the 10N HCl fraction, and was gradually replaced by di-oxidized spermine which had been bound to a serum component (▲) and whilst on the column had been dissociated the the acid solution. Unbound di-oxidized spermine (●) eluted in the 6N HCl fraction. Basic aldehyde$^{1+}$ material (□) eluted in the 4N HCl fraction. Neutral-charge material (×) eluted in distilled water fraction and probably represented by-products of labelled $H_2O_2$ and/or $NH_3$ with trace amounts of acrolein. Note that di-oxidized spermine would retain half-total [$^3$H] label from substrate (Table I) and therefore in serum-bound form represented maximally at 2–3 h incubation an equivalent of about 88% of substrate. Broken-lines represent extrapolations based on existing data.

FIG. 4.—Example of [$^3$H]-spermine (6.0 $\mu$M) conversion by 4·0 $\mu$g PAO in phosphate-buffer assay mixture. Unlike when FCS was used (Fig. 3) the complete exhaustion of the substrate (△) could be followed. This converted to a relatively small amount (7–14% substrate equivalent) of condensed or uncharacterized material (▲). Fractions and symbols are similar to those in Fig. 3. No other gross difference between FCS and purified PAO activities were discernible. Broken lines are extrapolations from existing data.

The di-oxidized spermine was formed within about 2 h incubation, but thereafter was destroyed with a half-life of about 2·3 h. When purified PAO replaced FCS as the source of enzyme in the assay mixture (*i.e.* either medium or PBS), there was an altogether unexpected finding (Fig. 3 and 4). When FCS had been used there was no apparent exhaustion of substrate. This was an artefact due to the di-oxidized spermine rapidly binding to a serum component. Obviously, such binding was not evident when purified PAO was used in PBS (Fig. 6). The complex dissociated by treatment with 10N HCl; by coincidence this was also the concentration required to release substrate from the ion-exchanger resin. The serum-binding component has yet to be identified. Gel-filtration (Sephadex G.200) indicated a mol. wt of roughly 70,000 for the complex. Quantitatively, the maximal bound di-oxidized spermine concentration was calculated to represent an equivalent of 88% of added substrate at pH 5·5–7·2 of reaction mixture at 2–4 h of incubation. The remainder must have comprised unbound, unreacted, unidentified and autodegraded material. The possibility of a rapid conversion of di-oxidized spermine to a further product that binds to serum cannot be discounted.

The nature of the decomposition compounds of di-oxidized spermine is unknown, and was not indicated in our study. There was no evidence for the for-

FIG. 5.—Examples of [$^{14}$C]-spermine (6·0 $\mu$M) conversion by FCS in tissue-culture conditions. The substrate (△) eluted in the 10N HCl fraction, and was gradually replaced by di-oxidized spermine which had been bound to a serum component (▲) and whilst on the column had been dissociated by the acid solution. Unbound di-oxidized spermine (●) eluted in the 6N HCl fraction. Basic aldehyde$^{1+}$ material (□) eluted in the 4N HCl fraction. Neutral charge material (×) eluted in the distilled water fraction. Note that very little of this was formed in comparison to [$^3$H]-spermine substrate conversion (Fig. 3), showing that the probable $H_2O_2$ and/or $NH_3$-labelled by-products were formed from the terminal moiety of the spermine molecule derived from the methionine grouping (Table I). Moreover there was little evidence of labelled acrolein being formed by autodegradation of di-oxidized spermine. Broken lines are extrapolations from existing data.

FIG. 6.—Comparison of [3H] di-oxidized spermine binding to a serum component in FCS and its relative absence in purified PAO buffer solution. Conversion of [3H]-spermine (6·0 µM) by FCS in tissue-culture medium, showing conversion of substrate (▽) to much serum-bound di-oxidized spermine (▼). In contrast, such conversion by 4·0 µg of PAO in buffer solution, showing conversion of substrate (△) to a small proportion of unidentified material$^{2+}$ (▲). Data were re-drawn for clarity from Fig. 1 and 2. The arrow indicates corrected curve for substrate decline after compensation for contamination in the fraction by serum-bound di-oxidized spermine. Similar results were obtained for [14C]-spermine conversion (not given). Broken lines are extrapolations from existing data.

mation of significant amounts of putrescine$^{2+}$, though other probable by-products of [3H]-spermine oxidation as well as decomposition, such as $NH_3$ and $H_2O_2$ (Tabor *et al.*, 1964), which together should retain half-label, were apparently formed but not positively identified (Fig. 3 and 4). The work of Kimes & Morris (1971) suggested a carbonyl$^{1+}$ derivative from autodegradation which was capable of condensing to oligamines$^{2+>3+}$. Acrolein is a carbonyl, but carries a neutral charge, and acrolein content was estimated by the colorimetric assay. It represented only 0·5% of substrate equivalent at the height of inhibitory activity and was insufficient to inhibit proliferation.

Curiously, when massive amounts of purified PAO were used in the assay mixture, there was further and complete conversion of di-oxidized spermine to a neutral fraction. This eluted from the ion-exchanger with distilled water, as shown in Fig. 7. It was positive for the aldehyde test, and would include acrolein. It is referred to as the neutral aldehyde fraction in Figs. 4–7. Furthermore, the material must be derived from the "[14C]-putrescine" moiety of the spermine molecule, since it incorporated all the radioactivity. The rate of the overall reaction converting spermine to neutral aldehyde by PAO (Fig. 7) was similar to the expected rate of conversion of benzylamine to benzaldehyde, used as the standard to

FIG. 7.—Conversion of [14C]-spermine by supra-physiological levels of purified PAO added to the assay mixture. Neutral aldehyde (○) production occurred to 100% substrate equivalent when a massive amount of purified PAO was used (1000 µg/500 mm³). The recovered material included acrolein, and was formed from the central "putrescine"-derived moiety of the substrate. Incubation period 14 h at 37°. (The graph failed to intersect at the origin because of a trace amount of a contaminant in the [14C]-spermine preparation.) Each point represents the mean of triplicate assays. The coefficient of variation was <5%.

obtain units of enzyme activity. The rate of formation of di-oxidized spermine was several hundred-fold faster.

Under culture conditions, a basic aldehyde[1+] fraction was also produced but only in small amounts equivalent to 5–10% of substrate. This was produced from both [$^3$H] and [$^{14}$C] labelled substrate, but the compounds carrying the labels need not be similar. The material from a large-scale preparation was separated chromatographically and dried on a rotary vacuum evaporator (40°C). No significant inhibitory activity was found in the preparation ($ID_{50}=500$ $\mu$g/cm$^3$). The extraction process, however, could well have destroyed it. The material had doubtful significance in the inhibitory system, since it was still present maximally at 24–38 h of incubation, when inhibitory activity had already waned.

PHA-treated lymphocytes, Bri8 and L5187Y lymphocytes, HP2 melanoma cells and V79 fibroblasts were somewhat variable in response to the inhibitory system (Table II). Retrospectively, variability was at least, in part, attributed to the contrasting kinetics of production and decomposition of oxidized polyamine pertinent to the cell cycle. Nevertheless, spermidine was much less effective than either spermine or bacterial thermine in the system (Table II), possibly because of its mono-oxidized structure.

### DISCUSSION

Interests that arose from our studies relate to the inhibitory system of polyamine oxidation by specific humoral enzyme (PAO) and whether it is physiological for *in-vivo* tissue-growth homeostasis, or even for malfunction in resolution and repair at sites of neoplastic growth, tissue damage and inflammation. The system has been implicated in immunoregulation (Byrd *et al.*, 1977; Allen *et al.*, 1977; Gaugas & Curzen, 1978). The phenomena of non-cytotoxicity, $G_1$-phase arrest of cell proliferation and DNA replication, as well as binding of di-oxidized spermine to a serum component, provides circumstantial evidence in support of some physiological or pathological role. Our findings suggest that di-oxidized spermine was the inhibitor, at least *in vitro*. If so, it reacts either in its free cationic form or when bound to an unidentified serum component. The latter is feasible partly because of the virtually instant binding of di-oxidized spermine. The importance of such binding requires elucidation. Hypothetically, $G_1$ arrest might reflect a property of the complex. Size of the complex (mol. wt 70,000) would preclude entry into a cell. It could attach to a surface-membrane receptor, however, which appears or functions only in the $G_1$ phase of the cell cycle. Alternatively, it could interfere with membrane receptors of vital growth factors (*e.g.* hormones) or transmembrane flux of $Ca^{2+}$, following initiation of the proliferation process. These possibilities are currently being investigated.

A cell would be capable of uptake of polyamine in its free oxidized cationic form. Intracellularly, the compound could compete with endogenous polyamine during its preparative role for mitosis, or by blocking polyamine biosynthesis. Polyamine biosynthesis occurs in the $G_1$ and $G_2$ phases of the cell cycle (see, for example, Fuller *et al.*, 1977). The polyamine analogue, methylglyoxal *bis*(guanylhydrazone), exerts its most potent effect from a diversity of pharmacological properties by inhibiting polyamine biosynthesis in a cell ($ID_{99}=1{\cdot}2$ $\mu$M). This produced $G_1$ arrest of proliferation, and was reversed by exogenous polyamines. Remarkably, the arrest was exerted not on progenitor cells but their daughter cells. Not surprisingly, therefore, it failed to inhibit transformation of PHA-stimulated lymphocytes (Gaugas & Chu, in preparation). This was because a cell contains a reserve pool of spermine for transfer to its daughters at mitosis (Newton & Abdel-Monem, 1978). Di-oxidized spermine did not, however, have such selectivity. In conclusion, there is no evidence to indicate the mechanism whereby oxidized polyamines arrest cell proliferation.

Most of the oxidation products of polyamines should contain the aldehyde grouping. Aldehydes are known to bind reversibly to the amino group of amino acids, to the basic residues of proteins, and to combine irreversibly with the sulphydryl groups of cysteine (Schauenstein *et al.*, 1977). Oxidized spermine has also been found to combine with nucleotides and DNA (Eilon & Bachrach, 1969). Polyamines in general are known to bind to many substances including glass, but there is also evidence of specific binding of spermine to phosphoprotein (Liang *et al.*, 1978). We have shown that the di-oxidized form also binds to an uncharacterized component in serum.

Urinary excretion of polyamines is greatly increased during malignancy, especially leukaemia. Urine levels may prove to be useful markers of disease activity (Russell & Durie, 1978). Similarly, PAO activity might be a useful indicator. Participation of the inhibitory system, together with binding of di-oxidized spermine, could possibly be of clinical importance in certain diseases involving tissue degeneration, in particular of the liver and kidney.

We thank the Cancer Research Campaign for support, Dr T. Oshima (Tokyo) for a gift of thermine, and Dr B. Thomas for L5187Y cells.

### REFERENCES

ALARCON, R. A. (1964) Isolation of acrolein from incubated mixtures of spermine with calf serum and its effects on mammalian cells. *Arch. Biochem. Biophys.*, **106**, 240.

ALARCON, R. A., FOLEY, G. E. & MODEST, E. J. (1961) Effects of spermine on mammalian cells. *Arch. Biochem. Biophys.*, **94**, 540.

ALLEN, J. C., SMITH, C. J., CURRY, M. C. & GAUGAS, J. M. (1977) Identification of a thymic inhibitor ("chalone") of lymphocyte transformation as a spermine complex. *Nature*, **267**, 623.

BACHRACH, U. & RECHES, B. (1966) Enzymic assay for spermine and spermidine. *Anal. Chem.*, **17**, 38.

BACHRACH, U, ABZUG, S. & BEKIERKUNST, A. (1967) Cytotoxic effect of oxidised spermine on Ehrlich ascites cells. *Biochim. Biophys. Acta*, **134**, 174.

BOYLAND, E. (1941) Experiments on the chemotherapy of cancer. 5. The effect of muscle extract and aliphatic bases. *Biochem. J.*, **35**, 1283.

BYRD, W. J., JACOBS, D. M. & AMOSS, M. S. (1977) Synthetic polyamines added to cultures containing bovine sera reversibly inhibit *in vitro* parameters of immunity. *Nature*, **267**, 621.

COHEN, S. S. (1977) Conference on polyamines in cancer. *Cancer Res.*, **37**, 939.

DEWEY, D. L. (1979) Methylglyoxal *bis*(guanylhydrazone) abolition of the toxic action of spermidine on cells in culture. *Cancer Letters* (in press).

EILON, G. & BACHRACH, U. (1969) Interaction of oxidised polyamines with DNA. *Biochem. Biophys. Acta*, **174**, 464.

FULLER, D. J. M., GERVER, E. W. & RUSSELL, D. H. (1977) Polyamine biosynthesis and accumulation during the $G_1$ phase of transition. *Cell. Physiol.*, **93**, 81.

GAUGAS, J. M. & CURZEN, P. (1978) Polyamine interaction with pregnancy serum in suppression of lymphocyte transformation. *Lancet*, i, 18.

HARDY, D. A. & LING, N. R. (1973) The mitotic activation of lymphocytes, biochemical and immunological consequences. In *The Cell Cycle in Development and Differentiation.* Eds I. M. Balls & F. S. Billet. Cambridge: University Press. p. 397.

HALEVY, S., FUCHS, Z. & MAGER, J. (1962) Toxicity of spermine and spermidine for tissue cultures. *Bull. Res. Council Israel*, **11A**, 52.

HEBY, O. & ANDERSSON, G. (1978) Tumour cell death. The probable cause of increased polyamine levels in physiological fluids. *Acta Pathol. Microbiol. Scand.*, **86** (Sect. A), 17.

HIGGINS, L. M., TILLMAN, M. C., RUPP, J. P. & LEACH, F. R. (1969) The effect of polyamines on cell cultures. *J. Cell. Physiol.*, **74**, 149.

HÖLTTÄ, E. (1977) Oxidation of spermidine and spermine in rat liver: purification and properties of polyamine oxidase. *Biochemistry*, **16**, 91.

ISRAEL, M. & MODEST, E. J. (1971) Synthesis of aminoethyl derivatives of $\alpha$-$\omega$-alkylene-diamines and structure-activity relationships for polyamine-bovine plasma amine oxidase system. *J. Med. Chem.*, **14**, 1024.

KAPELLER-ADLER, R. (1971) *Amine Oxidases and Methods for Their Study.* New York: Wiley.

KIMES, B. W. & MORRIS, D. R. (1971) Preparation and stability of oxidised polyamines. *Biochim. Biophys. Acta*, **228**, 223.

LIANG, T., MEZZATTI, G., CHEN, C. & LIAO, S. (1978) Selective polyamine-binding proteins. Spermine binding by an androgen-sensitive phosphoprotein. *Biochim. Biophys. Acta*, **542**, 430.

MAMONT, P. S., DUCHESNE, M.-C., GROVE, J. & BEY, P. (1978) Antiproliferative properties of DL-$\alpha$-difluoromethylornithine in cultured cells. A consequence of the reversible inhibition of ornithine decarboxylase. *Biochem. Biophys. Res. Commun.*, **81**, 58.

MELVIN, M. A. L. & KEIR, H. M. (1978) Diminished excretion of polyamines from BKH-21/C13 cells exposed to methylglyoxal *bis*(guanylhydrazone), *Biochem. J.*, **174**, 349.

NAIRN, R. C. (1969) *Fluorescent Protein Tracing.* London & Edinburgh: Livingstone. p. 306.

NEWTON, N. E. & ABDEL-MONEM, M. M. (1978) Inhibitors of polyamine biosynthesis. 4. Effects of $\alpha$-methyl-($\pm$)-ornithine and methylglyoxal *bis*-(guanylhydrazone) on growth and polyamine content of L1210 leukaemic cells of mice. *J. Med. Chem.*, **20**, 249.

Rijke, E. O. & Ballieux, R. E. (1978) Is thymus-derived lymphocyte inhibitor a polyamine? *Nature*, **274**, 804.

Russell, D. H. & Durie, B. G. M. (1978) *Polyamines as Biochemical Markers of Normal and Malignant Growth*. New York: Raven Press.

Schauenstein, E., Esterbauer, H. & Zollner, H. (1977) *Aldehydes in Biological Systems*. Transl. by P. Gore. London: Pion.

Tabor, C. W. & Tabor, H. (1976) 1,4-diaminobutane (putrescine), spermidine and spermine. *Biochem. Rev.*. p. 285.

Tabor, C., Tabor, H. & Bachrach, U. (1964) Identification of the aldehydes produced by the oxidation of spermine with purified plasma amine oxidase. *J. Biol. Chem.*, **239**, 2194.

Tabor, C. W., Tabor, H. & Rosenthal, S. M. (1954) Purification of amine oxidase from beef plasma. *J. Biol. Chem.*, **208**, 645.